# Supplementary material for: Cost-reducing adiabatic compressed air energy storage for long duration energy-storage applications
Source: iScience. 2025 Nov 7;28(12):113967. doi: 10.1016/j.isci.2025.113967 (PMC12677186; doi:10.1016/j.isci.2025.113967)
Supplement: Document S1. Figure S1 and S2, and Tables S2–S5 [file mmc1.pdf]

iScience, Volume *28*

Supplemental information

Cost-reducing adiabatic compressed air energy storage for long duration energy-storage applications

Danlei Yang, Yang Wang, Jihong Wang, Zhenhua Rui, and Wei He

Supplemental Information

This supporting information section has five components: (1) Two figures about the power cost and energy cost trends for CAES based on discharge duration with 60% and 70% RTE, (2) A table for original cost data for CAES projects with some assumptions, (3) A table for the validation of experience rate (ER), (4) A table for the parameters for discounted cash flow calculation, and (5) A table for the validation of the discounted cash flow approach.

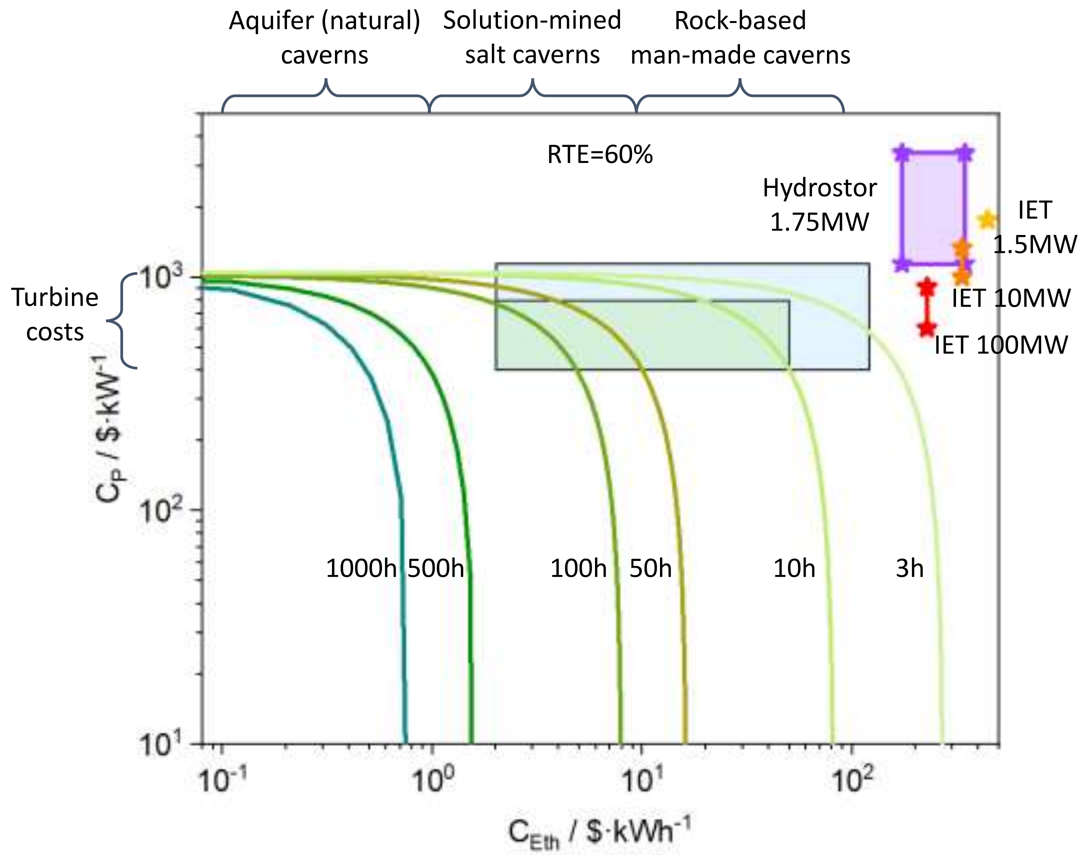

Figure S1 Power cost and energy cost trends for CAES based on discharge duration with 60% RTE.

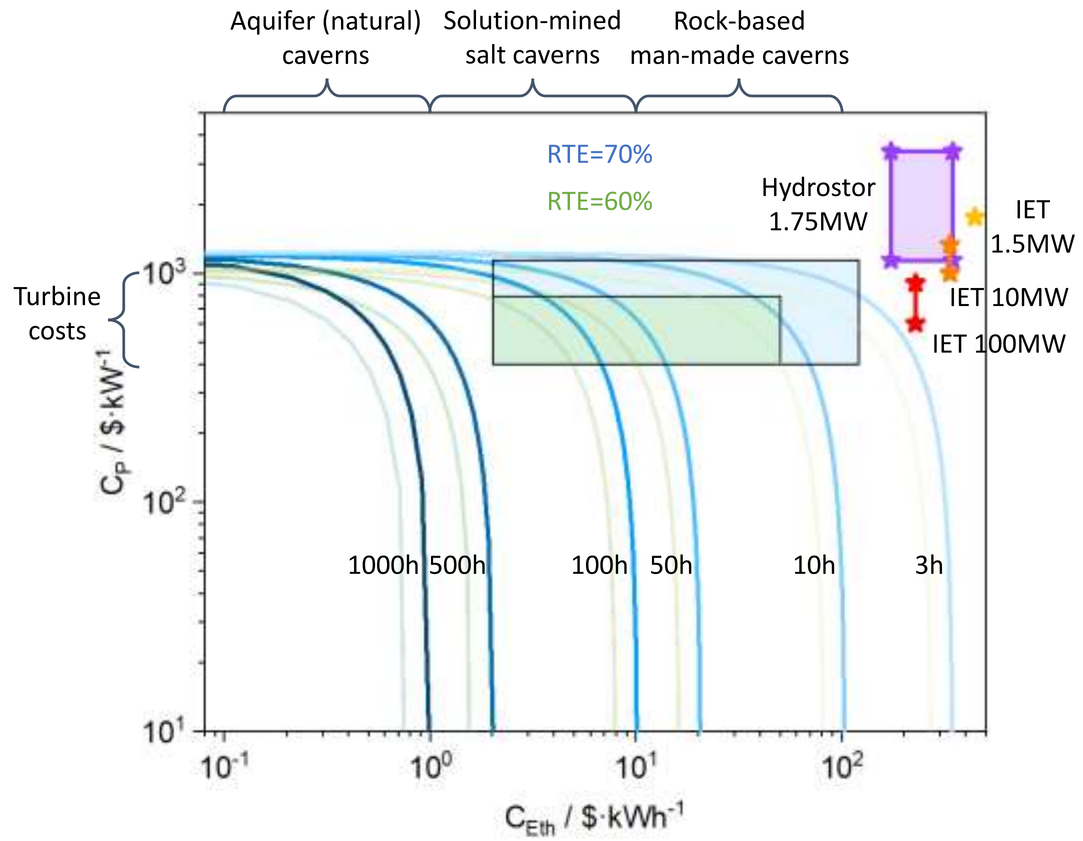

Figure S2 Comparison of power cost and energy cost trends for CAES at 70% and 60% RTE, based on discharge performance. The blue curves (RTE = 70%) are overall shifted to the right relative to the green curves (RTE = 60%), indicating that, for the same energy cost (C_Eth), the higher-efficiency system can achieve a lower power cost (C_P), or, for the same power cost, can accommodate a higher unit energy cost.

Table S2 Original cost data for CAES projects with some assumptions. C-rate is power-to-energy ratio.

Operation Date	Project	Power Capacity (MW)	Discharge Time (hours)	Capacity (MWh)	Investment	Investment Claim Year	Assumed Data
2013	Hebei Langfang	1.5	N/A	N/A	10k/kW (RMB)-2500 RMB/kWh	2013	C-rate=1/2
2014	Anhui Wuhu	0.5	1	0.5	30 million RMB	2012	N/A
2016	Guizhou Bijie	10	4	40	6-8k/kW (RMB)-2000 RMB/kWh	2016	N/A
2019	Goderich	1.75	5.71	10	1000-3000 $/kW	2019	N/A
2021	Shandong Feicheng (Phase 1)	10	N/A	N/A	1 billion RMB	2021	C-rate=1/4
2021	Jiangsu Jintan (Phase 1)	60	5	300	8333 RMB/kW	2021	N/A
2021	Hebei Zhangjiakou	100	4	400	8400 RMB/kW	2021	N/A
2024	Shandong Feicheng (Phase 2)	300	6	1800	1.5 billion RMB	2021	N/A
Future	Silver City	200	8	1600	600 million AUD	2022	N/A

Future	Henan Pingdingshan	200	N/A	N/A	7500 RMB/kW	2022	C-rate=1/8
Future	Hubei Yingcheng	300	5	1500	6000 RMB/kW	2022	N/A
Future	Shandong Taian (Phase 1)	350	4	1400	2.23 billion RMB	2022	N/A
Future	GEM	500	8	4000	335,959,300 $	2021	N/A
Future	Shandong Taian	2*300	N/A	N/A	5667 RMB/kW	2022	C-rate=1/8
Future	Jiangsu Jintan (Future Planning)	1000	5	300	55 billion RMB	2020	N/A
1978	Huntorf	290	2	580	116 million $	2002	N/A
1991	McIntosh	110	24	2640	45.1 million $	2002	N/A
2006	Huntorf	321	2	642	116 million $	2002	N/A

Table S3 Validation of the implemented learning rate model to estimate experience rate (ER). This table compares learning rates from our model with those reported in Schmidt et al., Nature Energy (2017). The strong agreement between modelled and published values validates the implemented model's ability to capture cost evolution in energy storage technologies. The uncertainty in the experience rate is estimated for the 95% confidence interval using the standard error, calculated as $\mu \pm 1.96\sigma$, where μ represents the mean and σ denotes the standard error of the tabulated experience rate.

EES technologies	Ref [S1]	Present	Uncertainty
Pumped hydro (utility)	-1±8%	0	[-12.90%, 8.33%]
Lead-acid (multiple)	4±6%	4%	[-1.56%, 10%]

Lithium-ion (utility)	12±3%	12%	[7.22%, 14.49%]
Sodium–sulfur (utility)	-	-	-
Vanadium redox-flow (utility)	11±9%	12%	[-12.9%, 31.79%]
Electrolysis (utility)	18±6%	19%	[9.52%, 27.59%]
Fuel cells (residential)	18±2%	21%	[18.79%, 22.79%]
A-CAES	-	15%	[10.03%, 19.55%]
D-CAES	-	-	

*Table S4 Parameters for discounted cash flow calculation. Some of the parameters are taken from Ref [S2], Figure 3, while others, not explicitly provided, have reasonable justifications. a. The number of annual full system cycles cannot exceed 4,380 hours divided by the duration time (d), since the total time in one year is 365 * 24 = 8,760 hours. One complete cycle consists of both charging and discharging, which takes 2 * duration time. Therefore, the maximum number of annual full system cycles is 8,760 divided by 2 * duration time, which equals 4,380/duration time. b. The values used for total capital cost were based on 70% of the maximum annual complete cycles for the given duration. c. A discount rate (r) of 7.5% is selected as it corresponds to the base case value in Ref [S2], Figure S1. d. The cycle life (L) is defined as the total project lifetime (T) multiplied by the number of equivalent complete cycles (n_c). e. FOM is set at 1% of the installed capital cost, representing a typical value for PSH [S3]. f. A 20-year period is chosen because, based on the validation of the discounted cash flow approach, it has been proven to be appropriate. g. The discharge efficiency (η_d) is assumed to be the square root of the round-trip efficiency (η_{RTE}).*

Parameter	Ref [S2], Figure 3.	Present
ΔE ($/kWh-cycle)	0.05	0.05
Annual full cycle	≤4380/d [a]	4380/d [a]
n_c (cycles/y)	70% Annual full cycle [b]	3066/d [b]
R_P ($/kW-y)	25	25
η_{RTE} (%)	50, 80	50, 80
r (%)	Not given	7.5 [c]

L (Cycle Life)	Not given	T*n_c [d]
P_C (\$/kWh-cycle)	0.025	0.025
FOM (\$/kW-y)	43.05 [e]	43.05 [e]
C_{Re} (\$/kWh)	0	0
VOM (\$/kWh-cycle)	0.002	0.002
d (hours)	10, 50, 100	10, 50, 100
T (years)	Not given	20 [f]
η_d (%)	Not given	Sqrt(η_{RTE}) [g]

Table S5 Validation of the discounted cash flow approach. This compares adjusted data from our model with the original data in Figure S1 of supplemental reference [S2]. Detailed comparison shows discrepancies generally below 10%, with high consistency between adjusted and original outputs further validating model reliability. Notably, the original document does not specify the parameter "T" (service life in years), so we assumed "T" to be 20 years for this analysis.

Parameter	Unfavourable Output	Unfavourable Output Adjusted	Error (%)	Base Output	Base Output Adjusted	Error (%)	Favourable Output	Favourable Output Adjusted	Error (%)
ΔE (\$/kWh-cycle)	7.61	7.78	2.27	20.35	20.53	0.86	33.09	33.27	0.54
n_c (cycles/y)	15.83	14.38	9.16	-	-	-	26.74	27.13	1.46
R_P (\$/kW-y)	15.25	15.43	1.17	-	-	-	25.45	25.62	0.68
η_{RTE} (%)	13.07	13.24	1.33	-	-	-	24.39	24.57	0.74
r (%)	17.47	17.59	0.69	-	-	-	24.09	24.35	1.09
Cycle Life	18.54	18.82	1.50	-	-	-	23.67	23.12	2.34
P_C (\$/kWh-cycle)	14.89	15.06	1.17	-	-	-	23.63	23.80	0.73
FOM (\$/kW-y)	18.31	18.49	0.96	-	-	-	22.39	22.56	0.78
C_{Re} (\$/kWh)	18.69	18.95	1.41	-	-	-	22.01	22.10	0.40
VOM (\$/kWh-cycle)	20.13	20.31	0.90	-	-	-	20.56	20.74	0.90

Supplemental References

[S1.] Schmidt O, Hawkes A, Gambhir A, Staffell I. The future cost of electrical energy storage based on experience rates. Nature Energy. 2017;2(8).doi:10.1038/nenergy.2017.110

[S2.] Albertus P, Manser JS, Litzelman S. Long-Duration Electricity Storage Applications, Economics, and Technologies. Joule. 2020;4(1):21-32.doi:10.1016/j.joule.2019.11.009

[S3.] Renewable energy technologies: cost analysis series-Hydropower. The International renewable energy agency. 2012
